# Importance of the delivery route for the tolerability and efficacy of immunomodulatory agents for lung cancer

**DOI:** 10.1038/s41598-026-58254-y

**Published:** 2026-06-19

**Authors:** Marion Ferreira, Aubin Pitiot, Christelle Parent, Eric Tartour, Damien Sizaret, Nathalie Heuzé-Vourc’h, Thomas Secher

**Affiliations:** 1 Service de pneumologie et explorations fonctionnelles respiratoires, Centre Hospitalier Régional Universitaire de Tours, Tours, France; 2 Centre d’Etude des Pathologies Respiratoires, U1100, INSERM, F-37032 Tours, France; 3 Faculté de Médecine, Université de Tours, F-37032 Tours, France; 4 PARCC (Paris Centre de Recherche Cardiovasculaire), INSERM U970, Université Paris Descartes, Paris, France; 5 Service d’Immunologie Biologique, Hôpital Européen Georges Pompidou, Paris, France; 6 Département d’anatomopathologie, Centre Hospitalier Régional Universitaire de Tours, Tours, France

**Keywords:** Lung cancer, Immunomostimulants, Airway administration, Toxicity, Immune response, Cancer, Immunology, Oncology

## Abstract

Although immune checkpoint inhibitors have revolutionized lung cancer treatment, their limited efficacy and significant toxicity highlight the need for improved therapeutic strategies. Immunomodulatory agents are known to enhance anti-tumor immune responses. In this study, we investigated how the route of administration affects the tolerability and immunological impact of these immunostimulants. We demonstrate that airway administration of poly I: C, CpG, and an anti-CD137 antibody in healthy mice results in significantly reduced liver toxicity and systemic inflammation compared with intravenous delivery. This was evidenced by lower AST levels, preserved body weight, and a reduced neutrophil-to-lymphocyte ratio. In addition, airway delivery of immunostimulant promoted dose-dependent recruitment of both myeloid and lymphoid cells as compared to intravenous administration. Using a syngeneic orthotopic mouse model of lung carcinoma, we further showed that local airway administration of anti-CD137 antibody resulted in increased pulmonary infiltration of CD4⁺, CD8⁺, and CD4⁺ TRM cells compared with intravenous injection. This enhanced immune infiltration correlated with improved tumor control. Overall, our findings indicate that local administration of immunostimulants enhances pulmonary adaptive immune responses and tumor suppression while limiting systemic toxicity.

**Supplementary Information:**

The online version contains supplementary material available at https://doi.org/10.1038/s41598-026-58254-y.

## Introduction

Lung cancer remains the leading cause of cancer-related mortality worldwide^1^. Despite significant therapeutic progress in recent years, it remains a major public health challenge, largely due to late-stage diagnoses, when metastases are often already present. This advanced stage is associated with high morbidity, mortality, and substantial societal costs. Over the past two decades, the therapeutic landscape of lung cancer has expanded with the advent of immune checkpoint inhibitors (ICIs), monoclonal antibodies that target inhibitory pathways restraining T lymphocyte activity. These agents have transformed the treatment of metastatic non-small cell lung cancer (NSCLC) by restoring anti-tumor immunity^2–4^. However, only 20–40% of patients benefit from ICIs, and their use may be accompanied by severe immune-related adverse events^5–9^.

As the indications for ICIs continue to broaden in NSCLC, considerable effort is being directed toward strategies that enhance their efficacy and overcome tumor immune evasion. Several factors can limit treatment response, including insufficient activation of anti-tumor CD8⁺ T cells, defective processing and presentation of neoantigens, the upregulation of alternative immune checkpoints, and the intrinsic heterogeneity of the tumor microenvironment^10–12^. In this context, cancer vaccines offer a promising approach to proactively prime the immune system to recognize and eliminate tumor cells by targeting tumor-associated antigens^13,14^. In lung cancer, therapeutic vaccination strategies encompass peptide-based, dendritic cell, and mRNA vaccines, often combined with ICIs to amplify anti-tumor responses^15,16^.

A critical determinant of vaccine or immunotherapy efficacy is the choice of adjuvant, which influences both the magnitude and quality of the immune response by engaging key immune cell populations. Several immunomodulatory agents have been explored, either alone or in combination with ICIs and vaccines^17^. In this study, we focus on three of them: CD137 agonistic antibodies, CpG oligonucleotides, and polyinosinic-polycytidylic acid (poly I: C). CD137 (4-1BB) is a costimulatory receptor of the TNF-receptor superfamily, and agonistic antibodies targeting CD137 preferentially expand CD8⁺ T cells and have shown the capacity to eradicate established tumors in murine models^18–20^. Notably, CD137 signaling has been implicated in the optimal accumulation of lung CD4⁺ and CD8⁺ tissue-resident memory T cells (TRM) following respiratory influenza infection^21^. The presence of these long-lived, non-circulating T cells has been shown to protect against tumor progression in preclinical models^22,23^ and to correlate with improved clinical outcomes in NSCLC, particularly in response to ICIs^24,25^. Another approach to potentiate anti-tumor immunity involves the activation of innate immune pathways via pattern recognition receptors, particularly Toll-like receptors (TLRs)^26^. CpG oligodeoxynucleotides, that mimic bacterial DNA, are recognized by TLR9 expressed on B cells and plasmacytoid dendritic cells^27^. In preclinical models, intratumoral injection of CpG can effectively control tumor growth by coordinating innate and adaptive immune responses^28^. Additionally, intranasal CpG administration has been shown to induce pro-inflammatory cytokines, including IL-1β, IL-6, and IL-8, in adults with allergic rhinitis^28^ and can drive the maturation of human monocytes into active dendritic cells within days^29^. Poly I: C, a synthetic analog of double-stranded RNA, activates TLR3 and MDA5, promoting the production of type I interferons and inflammatory cytokines to elicit potent antiviral and inflammatory responses^30^. In tumor-bearing mice, poly I: C treatment has been associated with reduced lung tumor growth through the establishment of a Th1- and Th17-like microenvironment^31^. In human glioblastoma, poly I: C-based vaccination elicited antigen-specific CD4⁺ and/or CD8⁺ T-cell responses in 75.4% of patients, with the majority demonstrating reactivity to multiple glioma-associated antigens^30^. Despite these promising immunomodulatory effects, the clinical application of immunomodulators often constrained by their toxicity profiles. CD137 agonists can induce dose-limiting hepatotoxicity, while poly I: C is known to provoke intense neutrophilic inflammation^32,33^.

An aspect of cancer immunotherapy that has received comparatively little attention is how the route of administration affects both the tolerability and therapeutic efficacy of immunomodulators. Currently, ICIs in NSCLC are administered intravenously (i.v.) or subcutaneously (s.c.), while vaccines are typically delivered intramuscularly or subcutaneously, with intranasal delivery gaining recent interest. However, systemic routes may not be optimal for lung cancer, where tumor antigens are localized within the lung tissue. This is especially true for large molecules like antibodies, for which only a small fraction of systemically administered doses reaches the airways^34,35^. Several studies have shown that TLR agonists such as CpG and poly I: C can enhance local innate and adaptive immune responses when delivered via the airways^36–38^. This route has been investigated in infectious disease models and, increasingly, in cancer, where localized immune activation may achieve superior tumor control while mitigating systemic toxicity^39^. Current efforts aim to refine dosing strategies and formulations to balance efficacy with the risk of inflammation-related adverse effects.

Given the importance of anatomical targeting in optimizing immunotherapy outcomes, we investigated how the route of administration modulates the pharmacodynamic and tolerability profiles of three immunomodulatory agents in healthy animals. We demonstrate that airway administration enhances immunomodulatory activity while reducing systemic toxicity relative to i.v. injection. Finally, in a robust, orthotopic, syngeneic, immunocompetent mouse model of lung cancer, we show that airway delivery of CD137 agonist improves therapeutic efficacy compared to systemic administration.

## Materials and methods

### Mice

Adult male C57BL/6jRj (B6) mice (7 to 8 weeks old) were obtained from Janvier (France). All mice were housed under specific pathogen-free conditions at the PST Animaleries animal facility (France) and had access to food and water ad libitum. All animal experiments complied with current European legislative, regulatory and ethical requirements and were approved by the local animal care and use committee (APAFIS#27858-2020110212288148 v4).

### Administration of tumor cells

The luciferase-expressing Lewis lung cancer (LL/2-Luc; LLC in short) cells were obtained from ATCC (CRL-1642). Cells were cultured in DMEM culture medium (Gibco), supplemented with 10% FCS (fetal calf serum, Gibco) and 1% penicillin-streptomycin (Gibco), at 37 °C under an atmosphere of 5% CO2. When reaching 50% of confluence, cells were detached using trypsin-EDTA 0.25% (Gibco). A solution of 20 × 10^6^ cells/mL was prepared and just before intratracheal administration, 10 mM of EDTA was added to the cell solution.

Mice were anesthetized with isofluorane 4%, and an operating otoscope fit with intubation specula was introduced to both maintain tongue retraction and visualize the glottis. A fibre optic wire threaded through a 20 G catheter and connected to lighted stylet (Harvard Apparatus, France) was inserted into the mouse trachea. Correct intubation was confirmed using lung inflation bulb test and 50 µL of LLC cells (1 × 10^6^ cells) was applied using an ultrafine pipette tip. Immediately after, a volume of 300 µL of air was gently administered using a syringe, to promote distal dissemination of the cells in the airways.

In all experiments, animal morbidity, mortality and body-weight were monitored daily. For ethical reasons, moribund animals and those who have lost more than 25% of their initial body weight were sacrificed.

### Administration of immunomodulatory agents

High molecular weight PolyI: C (tlrl-pic) and Class B CpG (ODN1826) were purchased from Invivogen. Anti-mouse CD137 (4-1BB) Ab (IgG2a, clone 3H3, BE0239) and anti-rat IgG2a Ab isotype control (clone 2EA, BE0089) were purchased from BioXCell. All reagents were prepared in PBS1X (Gibco). Administration of immunomodulatory molecules was performed either by via the caudal vein (for the i.v. route) in a total volume of 100µL or intratracheally using a MicroSprayer® aerosolizer (Penn-Century, USA) in a total volume of 50µL, as described previously^40^. Healthy mice were treated with Poly I: C at 1, 10–50 µg per doses; CpG at 1 and 5 µg per doses; anti-CD137 Ab at 10, 50–100 µg per dose. These doses were selected based on in vivo data found in the literature. Animals were sacrificed 24 h after administration. In the lung cancer model, mice were treated with anti-CD137 at 50 µg per doses on day 9, 14 and 18 after tumor inoculation. Animals were sacrificed at day 23.

### Organ sampling and flow cytometry

Mice were euthanized by intraperitoneal injection of pentobarbital (150 mg/kg). The lungs were rinsed by right ventricular perfusion with 10 mL of 1X PBS (ThermoFisher). The lungs were photographed to characterize the presence of tumors, their size and position. The lungs were digested, in the presence of 125 µg/mL of Liberase (Sigma) and 100 µg/mL of DnaseI (Sigma), and homogenized by mechanical dissociation (GentleMACS, Miltenyi) as previously described^41^. Cells were counted using Trypan Blue (Sigma) and processed for surface labeling with several antibody panels staining for CD45, CD3, CD4, CD8, CD11c, CD11b, CD44, CD62L, NKp46, Ly6G, IA/IE and CD1d, MR1 and TCRg tetramers. Dead cells were stained with the LIVE/DEAD Fixable Aqua Dead Cell Staining kit (ThermoFisher) and acquired on a MACS Quant (Miltenyi Biotec) cytometer. Analyses were performed using Venturi-One.

### Histology and blood parameters

After euthanasia, the lungs were rinsed by right ventricular perfusion with 10 mL of 1X PBS (ThermoFisher) and the right lobe was fixed by oro-tracheal administration of 500 µl of 4% paraformaldehyde and then immersed in the fixative solution overnight. After embedding in paraffin, tissue sections (4 μm) from the frontal planes were prepared on slides. Lung slides were stained with hematoxylin–eosin (H&E) for routine histology.

After euthanasia and before lung removal, bood was collected from the right ventricle using a 25 G needle (approximately 500 µL). Blood samples were homogenized and processed within one hour of collection with the IDEXX ProCyte Dx automatic hematology analyzer for veterinary use. The following parameters were obtained: red blood cell count (RBC, M/ µL), hematocrit (HCT, %), hemoglobin concentration (HB, g/dL), white blood cell count (WBC, K/µL), WBC differential total count and percentage of neutrophils (NEU, % and K/µL), lymphocytes (LYM,% and K/µL), monocytes (MONO, % and K/µL), eosinophils (EOS, % and K/µL) and basophils (BASO,% and K/µL). Neutrophil-to-lymphocyte ratio (NLR) was calculated by taking the ratio of the absolute neutrophil count to lymphocyte count. Liver transaminase levels were quantified in plasma obtained after euthanasia, using AST and ALT activity assay kit (Sigma-Aldrich).

### Bioluminescence

D-Luciferin (VivoGlow®) was purchased from Promega. To image tumor cells in vivo, mice were injected i.p. with 100 mg/kg of luciferin prepared in 1XPBS. Thirteen minutes after the injection, the animals were anesthetized with isoflurane (2% in 2 l/min oxygen), and bioluminescence was measured using the IVIS Lumina® system (PerkinElmer). Images were acquired over 3 min. Regions of interest (ROIs) were drawn around the chest wall, and the total number of photons within each ROI were recorded. ROI size was held constant across all images. The mice were imaged over time as described above, and quantitative analyses of the light emission were performed using Living Image software. The proportion of mice presenting detectable versus non-detectable luciferase signal following treatment was assessed.

### Statistical analysis

Statistical evaluation of differences between the experimental groups was determined by using one-way analysis of variance (ANOVA) followed by Tukey’s post hoc test for multiple comparisons. Student’s *t*-test (two-tailed) was used for comparison between two groups. χ^2^ test was used to assess differences in contingency tables. All tests were performed with GraphPad Prism, Version 9 for Windows (GraphPad Software Inc., San Diego, CA, USA; www.graphpad.com). Data presents box-and-whisker plots displaying the median, minimum, maximum, and all data points. A *p* value < 0.05 was considered significant.

## Results

### Systemic response and tolerability of immunostimulants according to the route of administration

**Fig. 1:**
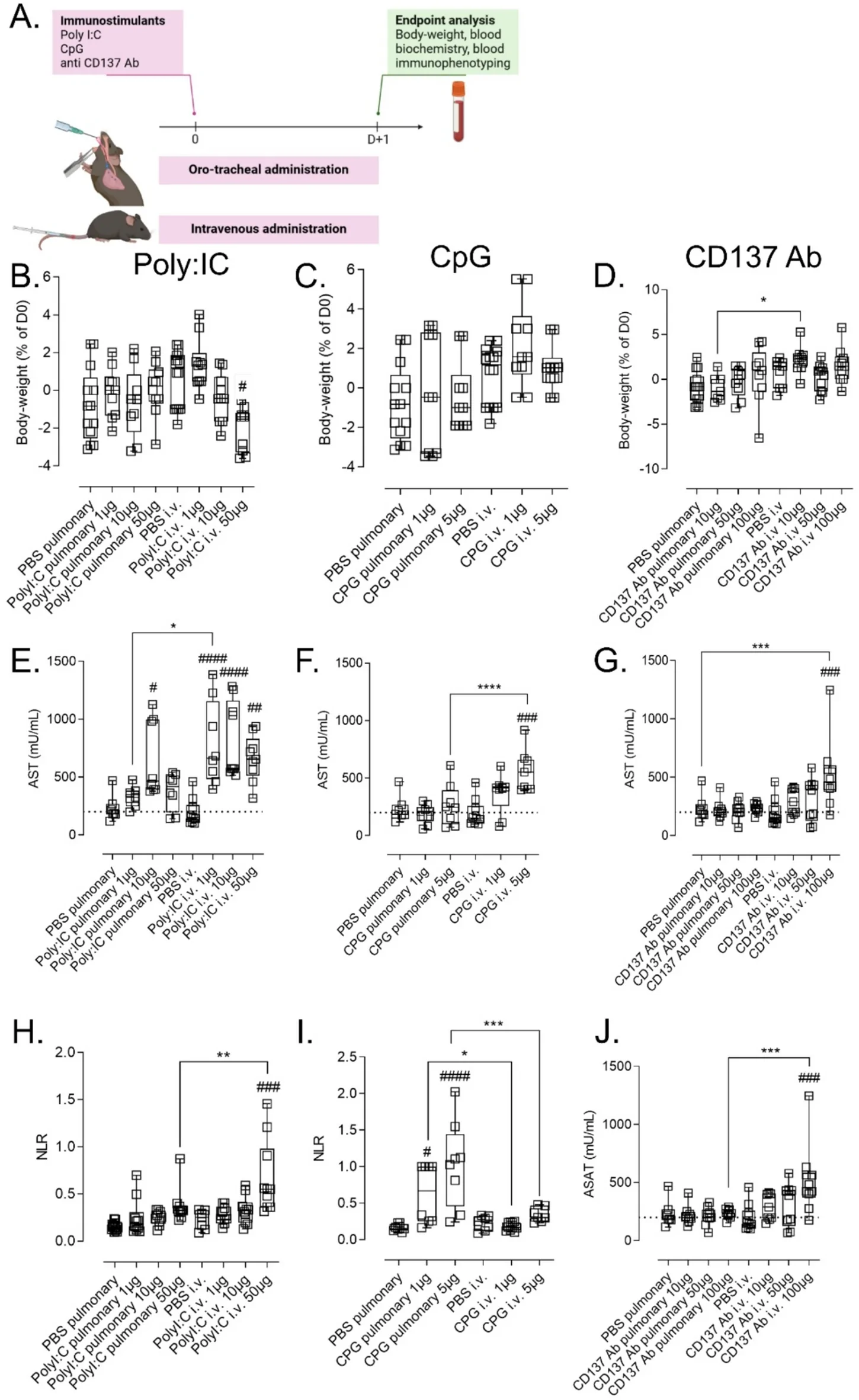
Systemic toxicity of immunomodulators according to their administration route. (**A**) C57/BL6jRj (B6) mice were treated or not with 1, 10–50 µg of Poly I: C; 1–5 µg of CpG; 10, 50–100 µg of anti-CD137 Ab in 50µL via the pulmonary (airway) route or in 100µL via the intravenous (systemic) route. Mice were sacrificed 24 h after the treatment. (**B**, **C** and **D**) Body-weight were monitored and expressed as % of the initial one. (**E**, **F** and **G**) Aspartate aminotransferase was determined in the serum using colorimetric assay. (**H**, **I** and **J**) Neutrophil-to-lymphocyte ratio was calculated based on the complete blood count realized on whole blood. The results correspond to 3 pooled, independent experiments (*n* = 15 mice per group) for each immunomodulators. *: *p <* 0.05; **: *p <* 0.01; ***: *p <* 0.001; ****: *p <* 0.0001 within immunomodulators. #: *p <* 0.05; ###: *p <* 0.001; ####: *p <* 0.0001 between immunomodulators and their respective control. Each statistical evaluation was made using a two-way ANOVA followed by a Tukey’s post-test.

We compared standard clinically used parenteral administration (intravenous, i.v.) with local airway delivery via intratracheal instillation to assess the impact of poly I: C (10, 50, and 100 µg), CpG (1 and 5 µg), and anti-CD137 antibody (10, 50, and 100 µg) on body weight and blood parameters in healthy mice at day 1 post-administration (Fig. 1A). The biosafety profiles of the immunostimulants were evaluated by monitoring body weight as an indicator of overall health status, and serum aspartate aminotransferase (AST) and alanine aminotransferase (ALT) levels as markers of hepatic tolerance. Intravenous administration of poly I: C at 50 µg induced a significant reduction in total body weight compared with PBS-treated controls, whereas local airway administration had no detectable effect (Fig. 1B). No significant changes in body weight were observed following CpG or anti-CD137 antibody administration, as compared to PBS-treated controls. Only a minor fluctuation was observed after the 10 µg dose of anti-CD137 antibody according to the route of administration (Fig. 1C–D). Intravenous injection of poly I: C resulted in a significant increase in AST levels at all tested doses. In contrast, pulmonary delivery of equivalent poly I: C doses led to markedly lower AST elevations, indicative of reduced hepatic toxicity (Fig. 1E). Similarly, at the highest dose tested, pulmonary administration of CpG was associated with significantly lower AST activity compared with systemic delivery (Fig. 1F). Finally, systemic administration of anti-CD137 antibody at the highest dose resulted in a significant increase in AST levels relative to PBS controls or the equivalent dose administered via the airway route (Fig. 1G). Additional blood biochemistry analyses revealed no further alterations following poly I: C, CpG, or anti-CD137 antibody treatment in healthy animals (Supplementary Fig. 1).

Because immunostimulants can influence systemic immune activation, we next quantified circulating neutrophils, lymphocytes, and monocytes using an automated hematological analyzer. Intravenous administration of poly I: C at 50 µg significantly increased the neutrophil-to-lymphocyte ratio (NLR) compared with PBS-treated controls and animals receiving the same dose via the airway route (Fig. 1H). Similarly, CpG administration induced a dose-dependent increase in NLR compared with PBS control or i.v.-injected CpG (Fig. 1I). In contrast, anti-CD137 antibody did not significantly affect the NLR (Fig. 1J). Extended blood cell profiling revealed no additional changes in immune cell populations (Supplementary Fig. 2). No differences were observed between placebo groups administered locally or systemically (Fig. 1).

### Pulmonary innate immune responses to immunostimulants according to the route of administration

**Fig. 2:**
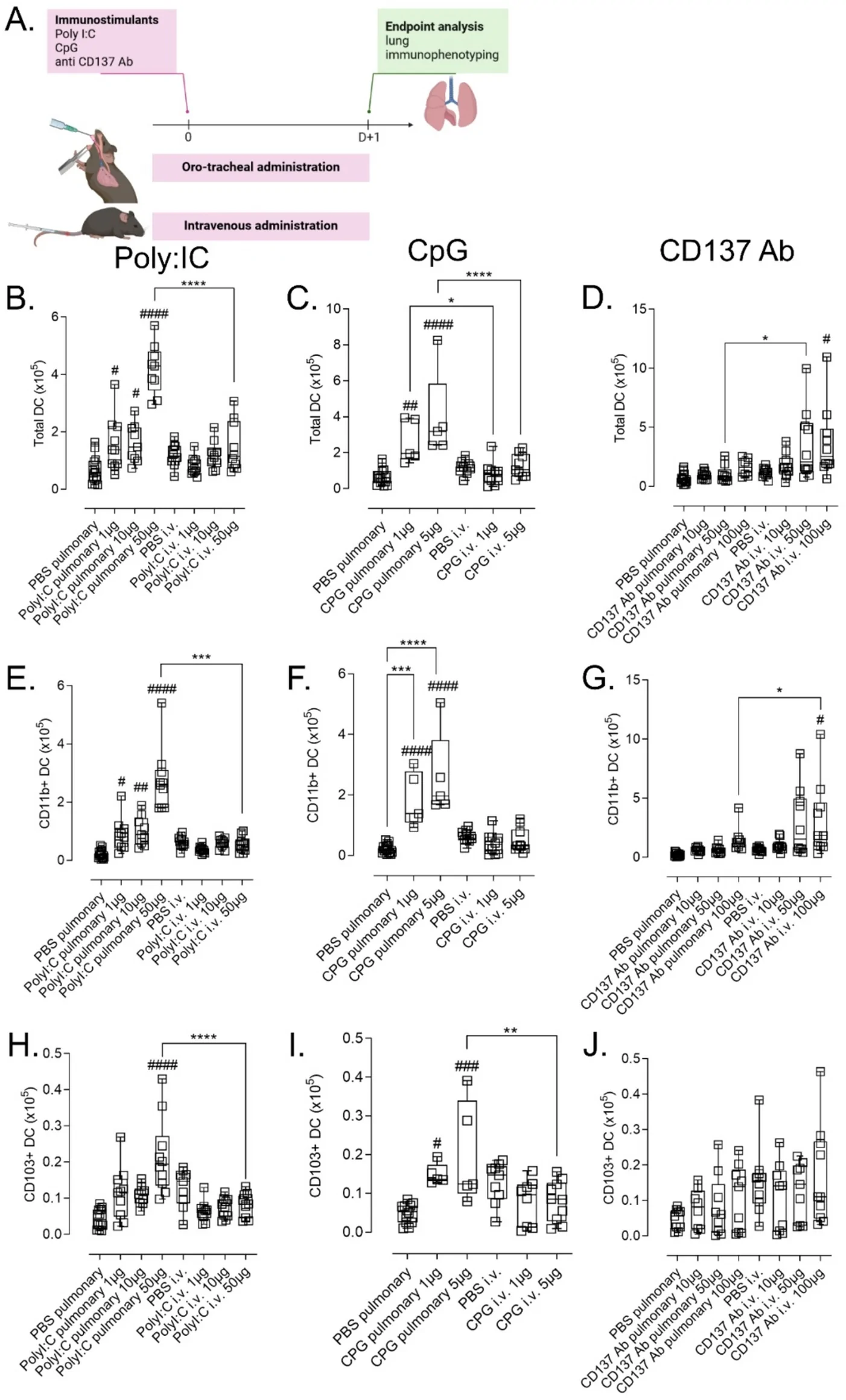
Impact of immunomodulators on lung dendritic cells according to their administration route. (**A**) C57/BL6jRj (B6) mice were treated or not with 1, 10–50 µg of Poly I: C; 1–5 µg of CpG; 10, 50–100 µg of anti-CD137 Ab in 50µL via the pulmonary (airway) route or in 100µL via the intravenous (systemic) route. Mice were sacrificed 24 h after the treatment. (**B**, **C**, **D**) Total conventional dendritic cells (CD45 + CD11c + MHC-II high) were quantified in whole lung homogenate using flow cytometry. (**E**, **F**, **G**) Conventional dendritic cells 2 (CD45 + CD11c + MHC-II high CD11b+) were quantified in whole lung homogenate using flow cytometry. (**H**, **I**, **J**) Conventional dendritic cells type 1 (CD45 + CD11c + MHC-II high CD103+) were quantified in whole lung homogenate using flow cytometry. The results correspond to 3 pooled, independent experiments (*n* = 15 mice per group) for each adjuvant. *: *p <* 0.05; **: *p <* 0.01; ***: *p <* 0.001; ****: *p <* 0.0001 within immunomodulators. #: *p <* 0.05; ##: *p <* 0.01; ####: *p <* 0.0001 between immunomodulators and their respective control. Each statistical evaluation was made using a two-way ANOVA followed by a Tukey’s post-test.

**Fig. 3:**
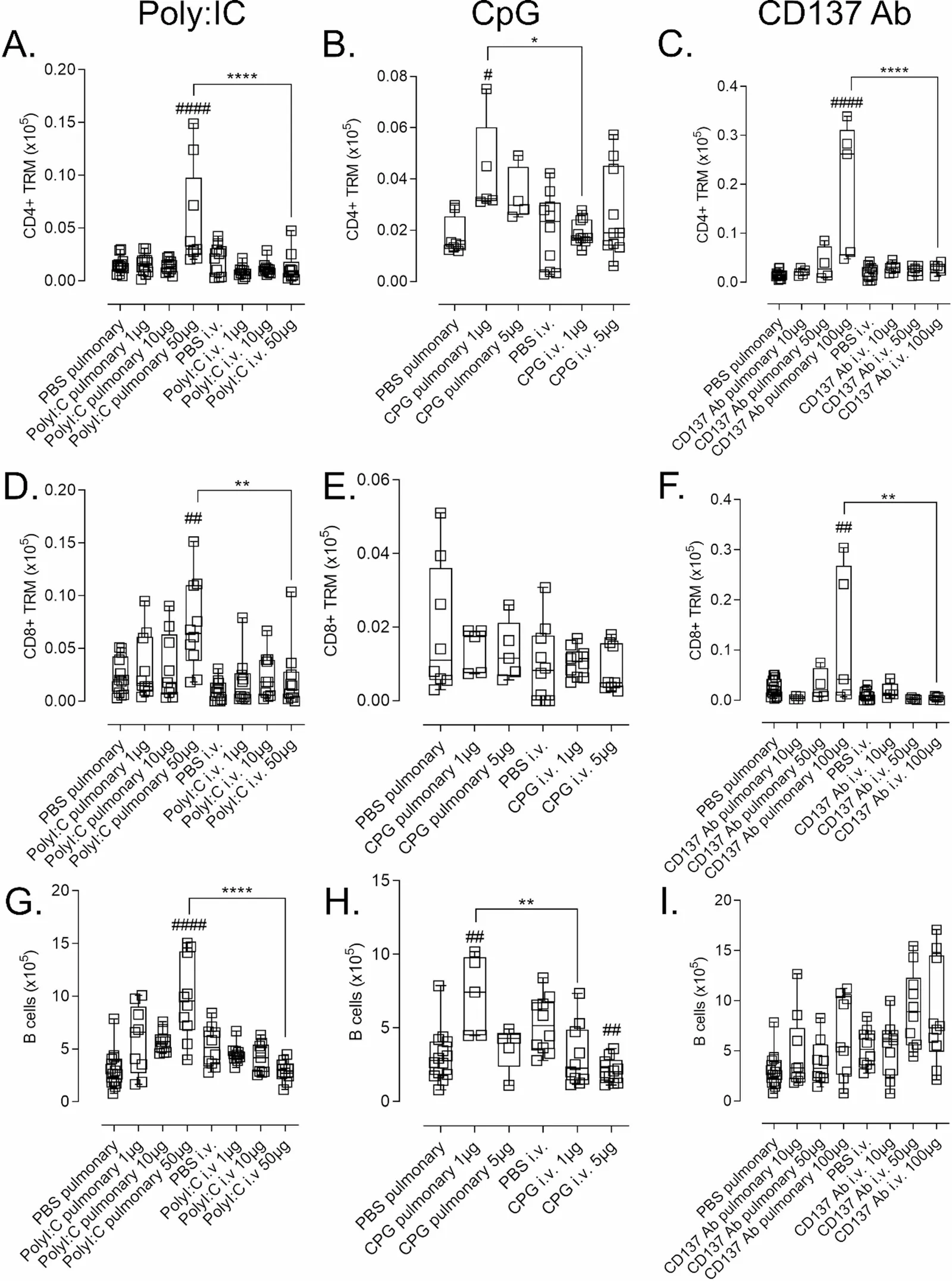
Impact of immunomodulators on lung lymphocytes according to their administration route. 24 h after treatment, (**A**, **B**, **C**) CD4 + T resident memory cells (CD45 + CD3+ CD4 + CD62L- CD44 + CD103+) were quantified in whole lung homogenate using flow cytometry. (**E**, **F**, **G**) CD8 + T resident memory cells (CD45 + CD3+ CD8 + CD62L- CD44 + CD103+) were quantified in whole lung homogenate using flow cytometry. (**G**, **H**,** I**) B lymphocytes (CD45 + CD3- CD19+) were quantified in whole lung homogenate using flow cytometry. The results correspond to 3 pooled, independent experiments (*n* = 15 mice per group) for each adjuvant. *: *p <* 0.05; **: *p <* 0.01; ***: *p <* 0.001; ****: *p <* 0.0001 within immunomodulators. #: *p <* 0.05; ##: *p <* 0.01; ####: *p <* 0.0001 between immunomodulators and their respective control. Each statistical evaluation was made using a two-way ANOVA followed by a Tukey’s post-test.

Local airway administration of poly I: C resulted in a marked, dose-dependent increase in total DCs, CD11b⁺ DCs, CD103⁺ DCs, CD4⁺ and CD8⁺ TRM cells, and B cells in the lung parenchyma compared with PBS-treated controls or equivalent doses administered intravenously (Figs. 2B,E,H and 3A,D,G). Similar increases were observed following pulmonary delivery of CpG (Figs. 2C,F,I and 3B,E,H). No differences were detected between placebo groups, irrespective of the route of administration (Fig. 2). In contrast, anti-CD137 antibody exhibited a distinct, route-dependent immunological profile. Intravenous administration of anti-CD137 antibody induced a dose-dependent increase in pulmonary dendritic cell populations compared with PBS controls or equivalent doses delivered locally (Fig. 2D,G,J).

### Pulmonary adaptive immune responses to immunostimulants according to the route of administration

Conversely to what observed in Fig. 2, airway delivery of anti-CD137 antibody led to a dose-dependent enrichment of both CD4⁺ and CD8⁺ TRM cells in the lung parenchyma relative to PBS controls or systemic administration (Fig. 3C,F), while no significant effect was observed on B-cell populations (Fig. 3I). These data highlight a route-specific modulation of pulmonary immune cell subsets following anti-CD137 antibody administration. No additional effects were detected on other pulmonary immune cell populations (data not shown). No differences were observed between placebo groups regardless of the administration route (Fig. 3).

### Establishment of an orthotopic mouse model of lung cancer

**Fig. 4:**
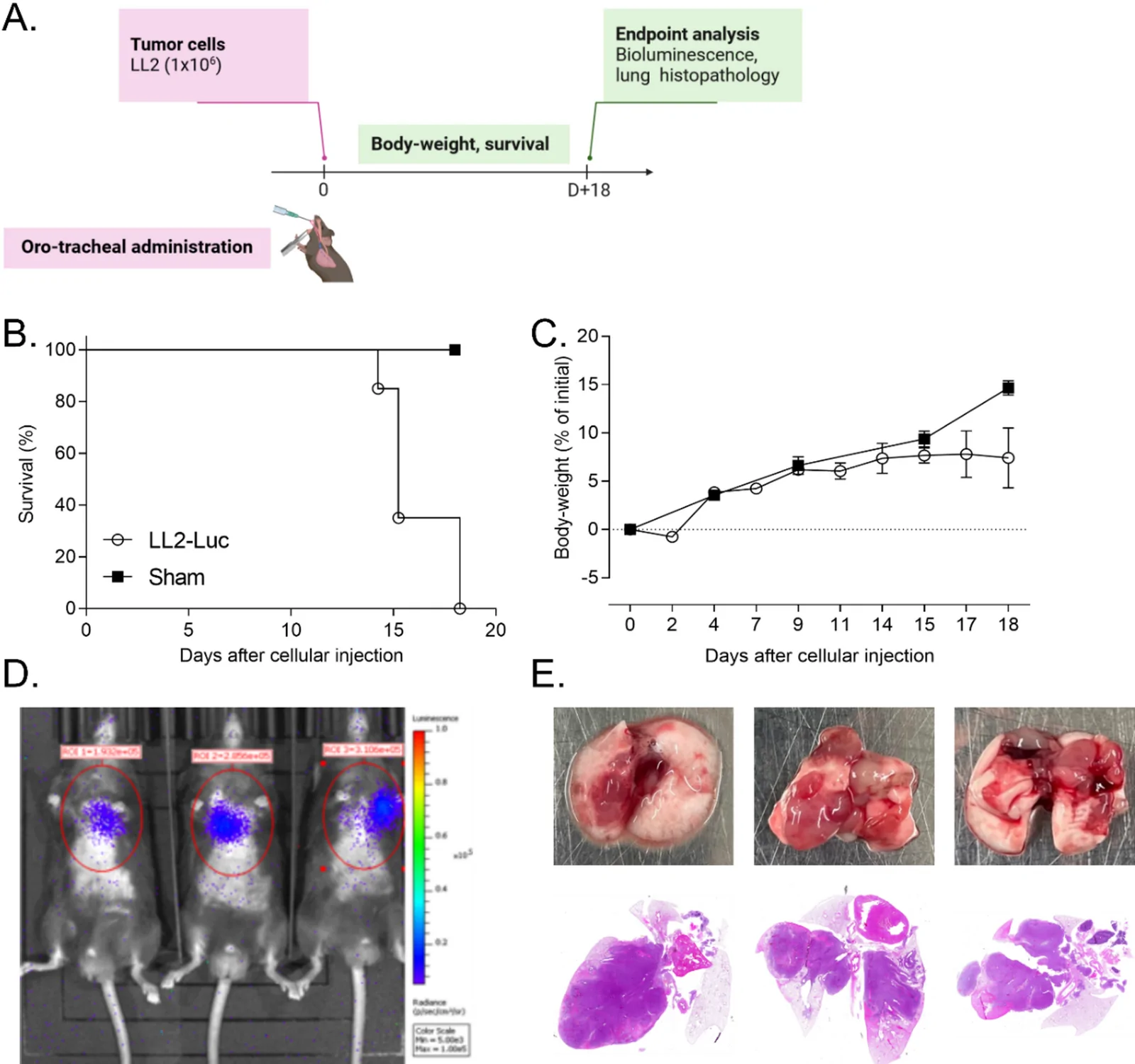
Characterization of an orthotopic mouse model of lung carcinoma (**A**) C57/BL6jRj (B6) mice were injected with 10^6^ LL2-*Luc* carcinoma cells (white square) or PBS1X (black square) in 50µL via the pulmonary (airway) route. Mice were sacrificed 18 days after cell inoculation. (**B**) Survival and (**C**) body-weight were monitored for 18 days. (**D**) 14 days after, tumor growth in the lung was monitored using bioluminescence (IVIS Lumina XR) in LL2-*Luc*-treated mice. (**E**) Gross and (**F**) histologic evaluation of whole lung and lung section stained with haematoxylin-eosin were performed at d + 18 after cell inoculation in LL2-*Luc*-treated mice. The results correspond to 4 pooled, independent experiments (*n* = 20 per group).

Having demonstrated that both tolerability and immunostimulatory activity are strongly influenced by the route of administration, we next established a relevant preclinical model to assess the therapeutic impact of immunostimulants. To this end, we developed an orthotopic mouse model of lung cancer, building on our expertise in pulmonary delivery of biologics and bacterial agents^41,42^. Immunocompetent mice were inoculated intratracheally with Lewis lung carcinoma (LL/2) cells (Fig. 4A). As shown in Fig. 4B, intratracheal administration of 1 × 10⁶ LL/2 cells resulted in mortality within 20 days. From day 14 onward, body weight stabilized, with no further significant increase observed as compared to sham (Fig. 4C). Tumor growth was monitored longitudinally using bioluminescence imaging. By day 14 post-inoculation, luminescent signals were consistently detected in all animals, confirming reliable tumor establishment within the lungs (Fig. 4D). Histological analysis of lung tissue using hematoxylin and eosin staining revealed the presence of intraparenchymal tumors displaying marked heterogeneity in size, anatomical distribution, and lateralization (Fig. 4E).

### Anti-tumor efficacy of anti-CD137 antibody monotherapy according to the route of administration

**Fig. 5:**
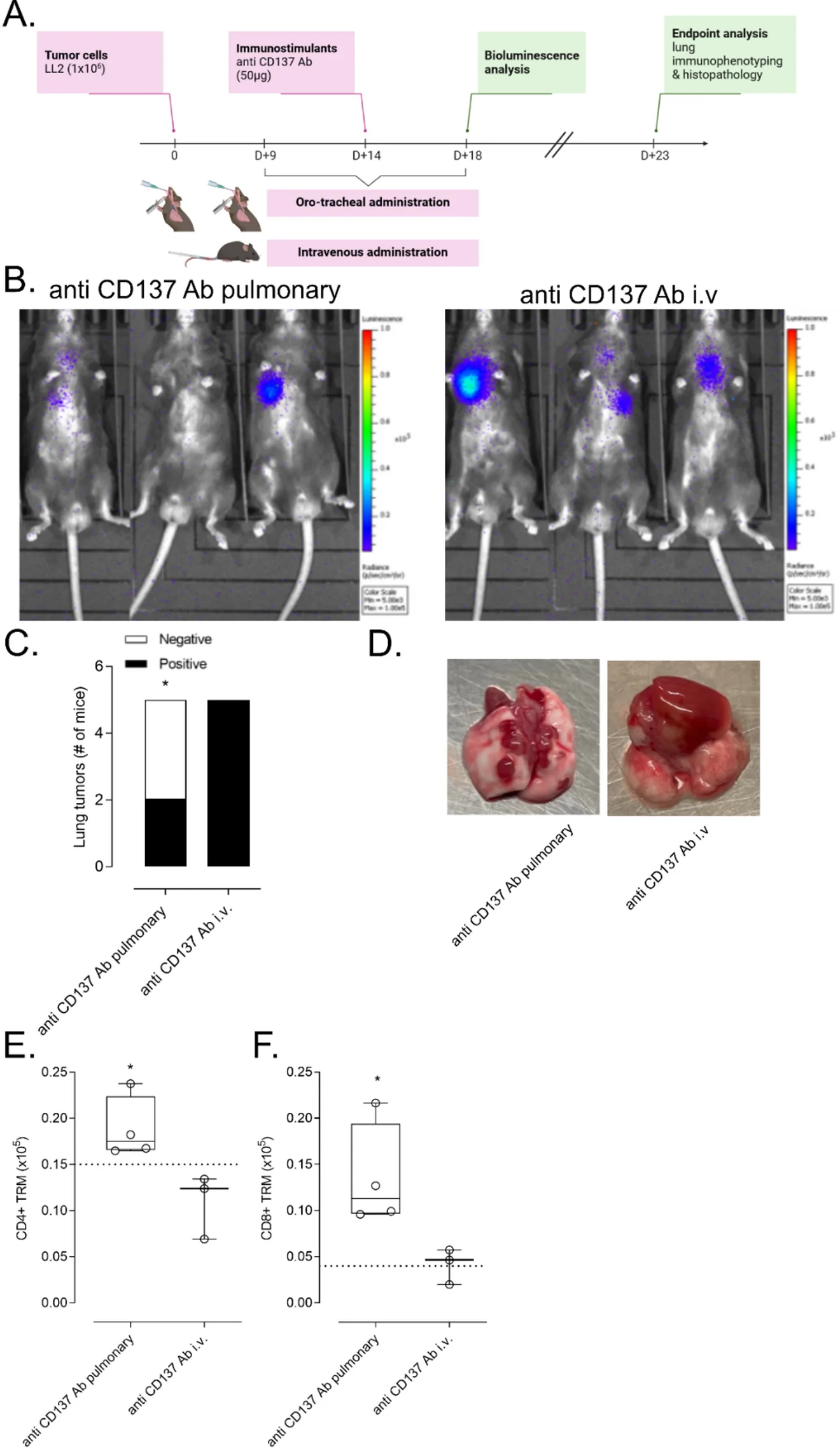
Anti-tumor effect of anti-CD137 Ab according to its route of administration (**A**) C57/BL6jRj (B6) mice were injected with 10^6^ LL2-*Luc* carcinoma cells in 50µL via the pulmonary (airway) route. At d + 9, d + 14 and d + 18 after cell inoculation, mice were treated with 50 µg of anti-CD137 Ab given either in 50µL via the pulmonary (airway) route or in 100µL via the intravenous (systemic) route. Mice were sacrificed at d + 23 after cell inoculation. (**B**) 18 days after cell inoculation, tumor growth in the lung was monitored using bioluminescence (IVIS Lumina XR) and mouse were classified as negative or positive according to the presence of detectable versus non-detectable luciferase signal (**C**). (**D**) Representative pictures of whole lung of mice treated with anti-CD137 Ab given through the airways (left) or i.v (right) at d + 23 after cell inoculation. (**E**) CD4 + and (**F**) CD8 + T resident memory cells were quantified in whole lung homogenate using flow cytometry at d + 23 after cell inoculation. Dotted represented the mean value for isotype-treated animals. The results correspond to 2 independent experiments (*n* = 8 per group). *: *p <* 0.05; with a χ^2^ test (**C**) or a Student’s *t*-test (**E** and **F**).

Based on the immunological findings described above (Figs. 2 and 3) and the observation of substantial pulmonary cell mortality following CpG and poly I: C treatment (data not shown), we focused subsequent therapeutic experiments on anti-CD137 antibody monotherapy in the syngeneic, orthotopic NSCLC mouse model. Given the enhanced pulmonary infiltration of CD4⁺, CD8⁺, and CD4⁺ TRM cells following airway administration of anti-CD137 antibody (Figs. 2 and 3), we evaluated a minimal effective dose of 50 µg delivered either intravenously or via intratracheal instillation at days 9, 14, and 18 after tumor implantation (Fig. 5A). Bioluminescence imaging revealed that local airway administration of anti-CD137 antibody significantly reduced tumor burden compared with systemic administration (Fig. 5B). Histological examination of lung sections further demonstrated reduced tumor infiltration of the pulmonary parenchyma in animals receiving local treatment relative to those treated intravenously (Fig. 5C,D). To elucidate the mechanisms underlying this therapeutic benefit, we assessed the presence of tissue-resident memory T cells within the lung parenchyma. Flow cytometric analysis revealed a significant increase in both CD4⁺ and CD8⁺ TRM cells in the lungs of tumor-bearing mice treated with locally administered anti-CD137 antibody compared with the i.v.-treated group (Fig. 5E,F) or the isotype-treated animals. Taken together, these findings indicate that localized delivery of anti-CD137 antibody to the lung compartment following tumor inoculation enhances pulmonary adaptive immune responses, which correlates with improved control of tumor growth.

## Discussion

A central question in the development of effective cancer immunotherapies is whether immunostimulatory agents can be combined or optimized to enhance anti-tumor efficacy^42–45^. The careful selection of immunostimulants is critical to elicit tuneable immune responses capable of reinforcing therapeutic outcomes. However, despite growing evidence that the route of administration can profoundly influence the magnitude, specificity, and safety of immune activation, this parameter remains insufficiently explored^46–48^. In the present study, we demonstrate that airway delivery of immunostimulants markedly enhances pulmonary immune responses while reducing systemic toxicity compared with intravenous administration. Using an immunocompetent orthotopic mouse model of lung cancer, we further show that airway administration promotes robust tissue-resident memory T (TRM) cell responses, which are associated with superior antitumor activity.

We first evaluated the tolerability of poly I: C, CpG, and anti-CD137 antibody according to their route of administration in healthy mice. Intravenous administration of poly I: C at 50 µg was associated with body weight loss and elevated aspartate aminotransferase (AST) levels, suggestive of hepatic stress. In contrast, pulmonary administration at the same dose resulted in minimal weight changes and significantly lower AST levels, indicating improved tolerability. A similar trend was observed for CpG and anti-CD137 antibody – except for one dose of the latter group which given the short time frame after administration may have no biologically meaningful effect. For both CpG and anti-CD137 antibody, systemic delivery increased markers of systemic toxicity compared with local airway administration. In addition, the neutrophil-to-lymphocyte ratio (NLR), a systemic inflammatory biomarker, was elevated following intravenous administration of poly I: C and CpG but remained unchanged after anti-CD137 antibody treatment. Importantly, elevated NLR has been associated with poorer clinical outcomes in patients receiving immunotherapy^49,50^. These observations are consistent with previous reports describing systemic toxicity following intravenous administration of poly I: C and CpG, including excessive pro-inflammatory cytokine release and liver injury^51–53^. In contrast, nasal or pulmonary delivery in other preclinical models has been shown to limit systemic toxicity by promoting localized immune activation while avoiding systemic cytokine storms^54–56^. Collectively, our findings suggest that local administration within the lung compartment represents a safer alternative to systemic delivery for immunostimulatory agents such as poly I: C, CpG, and anti-CD137 antibody. Nevertheless, our tolerability assessment was restricted to early indicators—including body weight, AST/ALT levels, and NLR—without long-term follow-up or histopathological analyses. The mechanisms underlying these route-dependent differences remain to be elucidated, underscoring the need for future studies addressing long-term safety and broader inflammatory consequences.

Beyond tolerability, we next investigated whether these distinct delivery routes also differentially shape pulmonary immune responses. Airway administration of poly I: C induced a dose-dependent recruitment of dendritic cells (DCs), including both CD11b⁺ and CD103⁺ subsets, as well as CD4⁺ and CD8⁺ TRM cells and B cells, compared with PBS-treated controls or equivalent systemic doses. Similar enrichment of adaptive immune populations was observed following pulmonary delivery of CpG. In contrast, anti-CD137 antibody exhibited a distinct route-dependent immunological profile: intravenous administration preferentially increased pulmonary DC recruitment, whereas airway delivery more effectively expanded lung-resident CD4⁺ and CD8⁺ TRM cells. B-cell numbers remained unchanged following anti-CD137 antibody treatment, and no significant alterations were detected in other immune cell subsets regardless of the administration route. Intratumoral DCs play a critical role in priming and sustaining antitumor T-cell responses through antigen presentation and effector T-cell activation within the tumor microenvironment (TME). Previous studies have shown that the presence of specific DC subsets, particularly CD103⁺ DCs associated with efficient cross-presentation, correlates with enhanced T-cell–mediated tumor control and improved clinical outcomes^57–60^. In the respiratory mucosa, although CD11b⁺ DCs populations have sometimes been associated with tolerogenic functions, they have been shown to participate in CD8⁺ T-cell priming, cytokine production, and amplification of adaptive immune responses continuously sample inhaled antigens and tumor-derived signals, orchestrating adaptive immune responses through antigen presentation and cytokine production^61–63^. Combining local adjuvant delivery with strategies that expand DC populations has shown promise in amplifying antitumor immunity. For instance, administration of Fms-like tyrosine kinase 3 ligand (FLT3L) to increase DC numbers, followed by localized poly I: C delivery, has been reported to enhance T-cell responses and promote tumor regression in preclinical models^64^. In lung cancer, mucosal DCs are optimally positioned to activate tumor-specific T cells locally, particularly TRM cells^65^. Whether anti-CD137 treatment directly modulates pulmonary DC subsets through CD137 signalling or indirectly through secondary immune activation remains to be determined. In this context, our data support the concept that local delivery of immunostimulants to the lung represents an effective strategy to initiate potent antitumor immune responses within the pulmonary microenvironment.

Having established route-dependent immune effects in healthy lungs, we then evaluated their relevance in a tumor-bearing setting, employing an orthotopic mouse model based on intratracheal instillation of LL/2 cells, a system widely validated for immunological studies in lung cancer^66–68^. Compared with intrathoracic or percutaneous injection approaches—which often require immunocompromised mice and entail surgical risks—this method offers a less invasive and immunologically intact alternative. Although genetically engineered mouse models better recapitulate certain aspects of human tumor complexity, they are often limited by prolonged latency, tumor heterogeneity, and metastatic dissemination^69,70^. Despite its limitations, including reliance on a single tumor cell line and the absence of spontaneous tumor initiation, the LL/2 model reproduces key features of NSCLC, such as peripheral lung tumor growth, stabilization of body weight from day 14 onward, and robust bioluminescent tumor signals.

Given its comparatively favorable toxicity profile relative to poly I: C and CpG, while retaining immunostimulatory capacity regardless of delivery route, we next assessed the therapeutic efficacy of anti-CD137 antibody in the orthotopic lung cancer model. Pulmonary administration of anti-CD137 antibody resulted in significantly improved tumor control compared with intravenous delivery, as demonstrated by bioluminescence imaging and histological analyses. This enhanced antitumor efficacy was associated with increased recruitment of lung-resident CD4⁺ and CD8⁺ TRM cells, indicating a strengthened local adaptive immune response. However, it is well established that the efficacy of immune checkpoint inhibitors depends on the pre-existing immune contexture of the tumor. In this regard, a study combining aerosolized hesperetin-loaded nanoparticles with an anti-CD40 antibody in a murine lung cancer model achieved complete tumor regression and immune memory in a subset of animals, highlighting the therapeutic potential of airway-delivered immunotherapy^71^.

These efficacy results prompted us to consider the immune mechanisms most likely mediating durable local tumor control. Sustained tumor control is increasingly thought to rely on targeting specific immune populations, particularly TRM cells^22,72^. Accumulating evidence suggests that the efficacy of immune checkpoint blockade depends on the presence of pre-existing T cells, especially tissue-resident populations^22,73^. Preclinical studies have shown that TRM cells contribute to durable immune surveillance and constrain tumor growth by maintaining an equilibrium between immune pressure and tumor escape^22,23^. Clinically, high densities of CD8⁺ TRM cells in NSCLC have been consistently associated with favorable prognosis^24,25^. Notably, a recent study demonstrated that only the intratumoral CD8⁺ TRM subpopulation co-expressing CD103 and CD49a is predictive of response to anti–PD-1 therapy in NSCLC patients, outperforming established biomarkers such as PD-L1 expression and TCF1⁺ CD8⁺ T-cell frequency in multivariate analyses^74^. In line with these observations, our results indicate that local administration of anti-CD137 antibody provides superior tumor control compared with systemic delivery, likely through enhanced recruitment and maintenance of lung-resident TRM cells. These findings are consistent with previous work demonstrating that airway delivery of the B subunit of Shiga toxin induces mucosal TRM responses and suppresses tumor progression, whereas intravenous administration is ineffective^75^.

Despite these encouraging mechanistic associations, several limitations should be acknowledged when interpreting the translational scope of our findings. Although our orthotopic model mainly reflects lung-confined disease rather than disseminated metastatic NSCLC, this setting was intentionally selected to investigate whether loco-regional delivery can optimize the therapeutic index of immunostimulatory molecules in the pulmonary tumor microenvironment. Importantly, local airway immunotherapy may still generate systemic anti-tumor responses through dendritic-cell activation, priming of tumor-specific T cells, and recirculation of activated lymphocytes^76,77^. However, systemic immune responses were not comprehensively characterized in the present work. While our data clearly demonstrate enhanced local pulmonary immunity, whether airway delivery induces durable systemic anti-tumor immunity remains to be established. Future studies should characterize peripheral immune responses and evaluate abscopal control in metastatic settings.

Nevertheless, these limitations do not preclude potential clinical applications of airway immunotherapy strategies. From a translational perspective, in the current therapeutic landscape of NSCLC, the most clinically relevant use of airway immunostimulants would likely be in combination with immune checkpoint inhibitors, administered via both local and systemic routes, rather than as monotherapy. Local activation of antigen-presenting cells and recruitment of effector T cells may enhance responsiveness to PD-1/PD-L1 blockade, particularly in poorly inflamed tumors^78^. Airway immunotherapy may also synergize with chemotherapy-based regimens. Several cytotoxic agents can induce immunogenic cell death, promote neoantigen release, and enhance antigen presentation, potentially amplifying the immune priming triggered by local pulmonary immunostimulation^79^. Combination studies are therefore warranted. These results support further clinical development of pulmonary immunotherapy approaches. From a practical standpoint, airway delivery could be implemented using currently available nebulization systems, dry-powder inhalers, or bronchoscopy administration strategies. Such approaches may allow high local drug exposure while limiting systemic concentrations^80^. However, several challenges remain, including heterogeneous aerosol deposition, airway obstruction, dose reproducibility, and careful monitoring of local inflammatory toxicity.

Taken together, these observations support further development of this therapeutic approach while highlighting key priorities for future investigation. Although a direct causal link between TRM expansion and tumor control in our model remains to be formally established, our data strongly support the therapeutic potential of pulmonary delivery of immunostimulatory agents for lung cancer treatment. Future studies using metastatic models will be required to determine whether pulmonary delivery may control extra-thoracic disease or synergize with systemic therapies. Additional work should assess long-term survival outcomes, explore combination strategies with immune checkpoint inhibitors, and further dissect the mechanistic contribution of TRM cells. Finally, incorporating TRM profiling into pre-therapeutic evaluations may help refine patient stratification and promote the development of mucosal immunotherapy approaches designed to induce protective tissue-resident immunity.

## Supplementary Information

Below is the link to the electronic supplementary material.

Supplementary Material 1

## Data Availability

The datasets used and/or analyzed during the current study available from the corresponding author onreasonable request.
