# Supplementary material for: Importance of the delivery route for the tolerability and efficacy of immunomodulatory agents for lung cancer
Source: Sci Rep. 2026 Jun 19;16:28127. doi: 10.1038/s41598-026-58254-y (PMC13554079; doi:10.1038/s41598-026-58254-y)
Supplement: Supplementary file 1 [file 41598_2026_58254_MOESM1_ESM.docx]

**Importance of the delivery route for the tolerability and efficacy of immunomodulatory agents for lung cancer**

Marion Ferreira^1,2,3*^, Aubin Pitiot^2^, Christelle Parent^2^, Eric Tartour^4,5^, Damien Sizaret^6^, Nathalie Heuzé-Vourc’h^2,3^, Thomas Secher^2,3^

**Figure legends**

Supplementary Figure 1: Hematological analysis

Whole blood was analyzed using Procyte, 24h after treatment with Poly I:C (A, B and C), or CpG (D, E and F) or anti-CD137 Ab (G, H and I). (A, D and G) Red blood cell number. (B, E and H) Hemoglobin concentration. (C, F and I). Hematocrit concentration. The results correspond to 3 pooled, independent experiments (n=15 mice per group) for each adjuvant. ***: *p<*0.001 within immunomodulators. #: *p<*0.05; ##: *p<*0.01 between immunomodulators and their respective control. Each statistical evaluation was made using a two-way ANOVA followed by a Tukey’s post-test.

Supplementary Figure 2: Complete blood count analysis

CBC was analyzed in whole blood using Procyte, 24h after treatment with Poly I:C (A, B and C), or CpG (D, E and F) or anti-CD137 Ab (G, H and I). (A, D and G) Neutrophil concentration in blood. (B, E and H) Lymphocyte concentration in blood. (C, F and I). Monocytes concentration in blood. The results correspond to 3 pooled, independent experiments (n=15 mice per group) for each adjuvant. ***: *p<*0.001 within immunomodulators. #: *p<*0.05; ##: *p<*0.01 between immunomodulators and their respective control. Each statistical evaluation was made using a two-way ANOVA followed by a Tukey’s post-test.

Supplementary Figure 3: Gating strategy for lung immunophenotyping

(A) Conventional dendritic cells type 1 were defined as Live CD45+ CD11c+ MHCII high CD103+ and conventional dendritic cell type 2 were defined as Live CD45+ CD11c+ MHCII high CD11b+.

(B) T resident memory cells were defined as either CD4+ or CD8+ Live CD45+ CD3+ CD62L- CD44+ CD103+.

Supplementary figure 1 :

Supplementary Figure 2:

Supplementary figure 3:
